# Normative Reference Values of the Tibial Nerve in Healthy Individuals Using Ultrasonography: A Systematic Review and Meta-Analysis

**DOI:** 10.3390/jcm12196186

**Published:** 2023-09-25

**Authors:** Thanyaporn Senarai, Athikhun Suwannakhan, Thongchai Pratipanawatr, Kaissar Yammine, Laphatrada Yurasakpong, Tanapat Sathapornsermsuk, Sirorat Janta, Achiraya Kittiboonya

**Affiliations:** 1 Microscopy Unit, Department of Anatomy, Faculty of Medicine, Khon Kaen University, Khon Kaen 40002, Thailand; thansen@kku.ac.th; 2 Department of Anatomy, Faculty of Science, Mahidol University, Bangkok 10400, Thailand; laphatrada.yur@mahidol.edu; 3 In Silico and Clinical Anatomy Research Group (iSCAN), Bangkok 10400, Thailand; 4 Department of Internal Medicine, Faculty of Medicine, Khon Kaen University, Khon Kaen 40002, Thailand; thongcha@kku.ac.th; 5 Department of Orthopedic and Trauma Surgery, Lebanese American University Medical Center—Rizk Hospital, Beirut 11-3288, Lebanon; cesar.yammine@laumcrh.com; 6 The Center for Evidence-Based Anatomy, Sports and Orthopedic Research, Lebanese American University, Byblos 1102-2801, Lebanon; 7 Mahidol University International College, Salaya, Nakhon Pathom 73170, Thailand; tanapat.sa@hotmail.com; 8 Anatomy Unit, Department of Medical Science, Faculty of Science, Rangsit University, Pathumthani 12000, Thailand; sirorat.j@rsu.ac.th; 9 Centre for Anatomy and Human Identification (CAHID), School of Science and Engineering, University of Dundee, Dundee DD1 4HN, UK; 2588636@dundee.ac.uk

**Keywords:** tibial nerve, ultrasound, sonography, reference values, systematic review, meta-analysis

## Abstract

Background: High-resolution ultrasound of the tibial nerve has been used for screening of several neurologic disorders, but normative reference values of tibial nerve cross-sectional areas (CSA) have not been well established. Thus, the present meta-analysis was performed to generate normative estimates of tibial nerve CSA at various sites of the lower limb based on ultrasonography. Methods: Google Scholar, Scopus and PubMed were searched for potential studies. Studies were required to report tibial nerve CSA in healthy individuals to be included. A random-effect meta-analysis was performed to calculate tibial nerve CSA values. Subgroup and statistical analyses were performed to study covariates. Results: Forty-eight eligible articles consisting of 2695 limbs were included. The average tibial nerve CSA was found to be 10.9 mm^2^ at the ankle (95% CI: 9.9–11.8) and should not exceed 11.8 mm^2^ in healthy adults. At the popliteal fossa, the overall CSA was 21.7 mm^2^ (95% CI: 17.5–25.8) in healthy adults. At both sites, the average tibial nerve CSA was significantly larger in adults than in children, and the differences by geographical region were not statistically significant. At the ankle, tibial nerve CSA increased with age and body mass index, while at the popliteal fossa it increased with age and weight. Conclusions: our findings indicate that the tibial nerve varied not only along its course but also among sub-variables. Establishing normal references values of tibial nerve CSA is helpful to differentiate healthy from diseased tibial nerves such as in diabetic peripheral neuropathy or tarsal tunnel syndrome.

## 1. Introduction

High resolution ultrasound is a powerful and cost-effective imaging modality for depicting peripheral nerves [1]. It clearly demonstrates the morphological changes to the nerve and its precise anatomical position [2], which complement electrodiagnostic studies. In addition, it is a trusted diagnostic tool for tracing and assessing peripheral nerves in several neurologic disorders, including compression neuropathies and chronic inflammatory neuropathies [3]. The most widely accepted measurement is the cross-sectional area (CSA), obtained by being positioned over the nerve at sites of interest. Determination of normal CSA values is crucial to differentiate between normal and abnormal nerves. This has become a subject of interest recently.

The tibial nerve is one of the two branches of the sciatic nerve, providing motor and sensory supplies to most part of the posterior compartment of the leg and foot. The increase in tibial nerve CSA has been implicated in neurologic disorders including diabetic peripheral neuropathy and tarsal tunnel syndrome [4,5,6,7,8,9,10,11]. Establishing normative data on tibial nerve CSA would be clinically helpful in differentiating between healthy and abnormal tibial nerves. A recent meta-analysis [12] has presented CSA values of the tibial nerve in healthy individuals, derived from 16 ultrasonographic studies. However, it is important to acknowledge that a significant number of studies may be absent from this analysis due to the omission of healthy control groups from studies that focused on tibial nerve CSA in patients with various diseases. Including CSA values of healthy controls from these studies would contribute to the establishment of more dependable reference values for the CSA of the tibial nerve. In addition, some authors have reported the effect of age [13,14], weight [15,16] or other parameters on tibial nerve CSA, whereas others [7,17] did not confirm any association. Previously established normal reference values have also varied widely, possibly because of other factors such as varying measurement levels, differences in the examined population and the use of different ultrasound systems [18]. Therefore, a more robust approach is needed to provide more reliable sonographic reference values of tibial nerve CSA.

The aim of the present study was to conduct a systematic review and meta-analysis of published CSA of the tibial nerve. This was undertaken in order to establish normal reference values and to identify potential factors that influence tibial nerve CSA in healthy individuals.

## 2. Materials and Methods

This systematic review and meta-analysis accorded with the PRISMA 2020 guidelines [19], the Checklist for Anatomical Reviews and Meta-analysis (CARMA) [20], and the Critical Appraisal Tool for Anatomical Meta-analysis (CATAM) [21]. The protocol employed in this study was registered on PROSPERO (CRD42020218941). Ethical approval was not required for this study as this is a non-interventional literature review and analysis of published articles from online databases.

### 2.1. Systematic Literature Search and Study Selection

**Figure 1:**
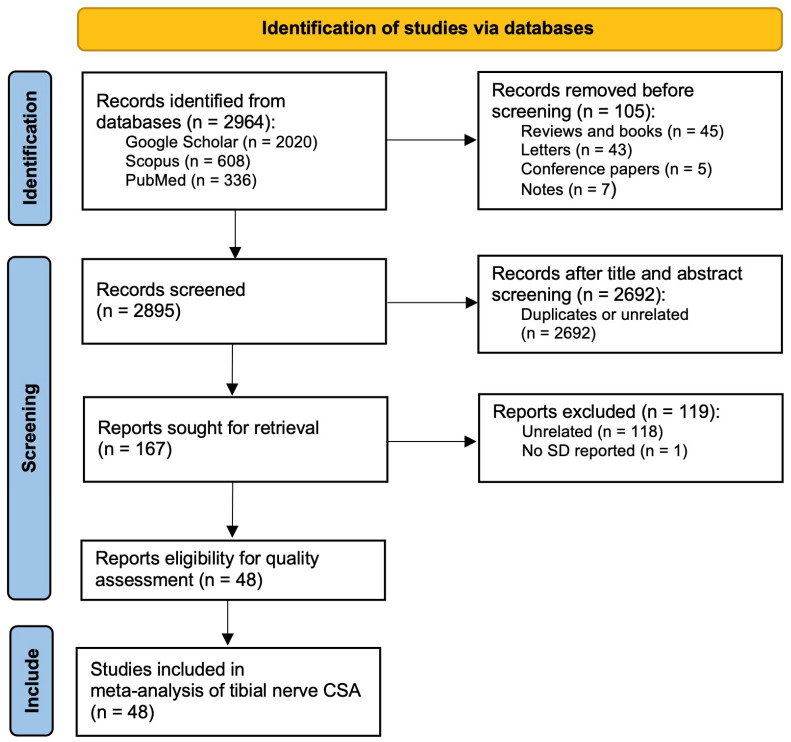
PRISMA flowchart showing the protocol of the present meta-analysis.

A systematic literature search was conducted as summarized in Figure 1. Searches were independently performed through Google Scholar, Scopus and PubMed. For Google Scholar, the following keywords were used: “tibial nerve” AND (“ultrasound” OR “sonography”) AND “cross-sectional area”. For Scopus and PubMed databases, the following keywords were used: “tibial nerve” AND (“ultrasound” OR “sonography” OR “cross-sectional area”). Document types including reviews articles, letters, book chapters and conference papers were excluded.

Study screening was conducted by two authors (T.Se. and A.S.), both holding PhDs and a strong background in radiologic anatomy. Author T.Se. has received training in ultrasound imaging from an independent expert radiologist with 15 years of experience in diagnostic imaging. The selection of studies was undertaken by these two authors (T.Se. and A.S.) in consultation with the third author (T.P.), a highly experienced endocrinologist with over three decades of expertise. Each study underwent a comprehensive examination (by T.Se. and A.S.) with meticulous attention to the methodology employed. Further investigation was conducted on studies that fulfilled the following criteria: (1) tibial nerve CSA was reported; (2) tibial nerve CSA was measured by ultrasound; (3) location of CSA measurement was reported; (4) number of subjects were reported; and (5) patients included were healthy/had no history of diabetes or poor vascular state. Studies that failed at least one of the aforementioned criteria were excluded from the analysis. More studies were excluded when the results were poorly or not clearly reported, for instance, if there was a lack of standard deviation or if they did not pass an assessment on the risk of bias. Factors that may influence tibial nerve CSA, including geographical region, age (children or adults), weight and body mass index (BMI), were investigated further by subgroup analysis or correlational analysis.

### 2.2. Risk of Bias Assessment

The quality of the potential studies was assessed using the Revised-Quality Assessment of Diagnostic Accuracy Studies (QUADAS-2) tool [22]. The QUADAS-2 tool was designed to evaluate four aspects of methodological quality including patient selection, details of the index test, description of the reference standard, and flow and timing of participant recruitment. Each item was rated by two authors as “low risk”, “high risk” or “unclear”. Any disagreement between the two assessors was resolved by a third.

### 2.3. Meta-Analysis

A random-effect meta-analysis was used to generate pooled-estimates of tibial nerve CSA. The data that were extracted include number of subjects, tibial nerve CSA, standard deviations, measurement location, country, age, weight and BMI. Measurement locations include sciatic nerve bifurcation, popliteal fossa, midcalf and ankle. When tibial nerve CSA was measured at the ankle, distance to the medial malleolus was recorded. The primary outcomes were average tibial nerve CSA in healthy individuals by locations. The secondary outcomes include subgroup analysis by geographical region, age group and BMI categories. Regression analysis was carried out to study the effect of age, weight and BMI on tibial nerve CSA. The effect sizes were reported with 95% confidence intervals and standard deviations. Standard errors were calculated using the equation SE = SD/√ (number of subjects). Between-study heterogeneity was assessed using I^2^ statistics. Within-subgroup differences were assessed using Q-statistics. Publication bias was evaluated using a funnel plot of effect sizes versus standard errors and Egger’s regression test.

Meta-analysis and all statistical analyses were performed using Stata version 17 (StataCorp, Lakeway, TX, USA). Statistical significance was established at *p* = 0.05 (two-tailed).

## 3. Results

### 3.1. Systematic Review

The systematic literature search yielded a total of 2020 entries on Google Scholar, 608 entries on PubMed, and 336 entries on Scopus (Figure 1). A total of 105 were initially excluded including 45 reviews and books, 43 letters, 5 conference papers and 7 notes. Abstract screening obtained a total of 167 potential studies. The full texts of these studies were downloaded and read thoroughly. One-hundred and twenty-seven entries were further excluded including 120 unrelated studies and 1 study in which standard deviation was not reported. In total, 48 studies met the inclusion criteria and underwent risk of bias assessment. Characteristics of these 48 studies and raw data for meta-analysis is available in File S1.

### 3.2. Risk of Bias Assessment

**Figure 2:**
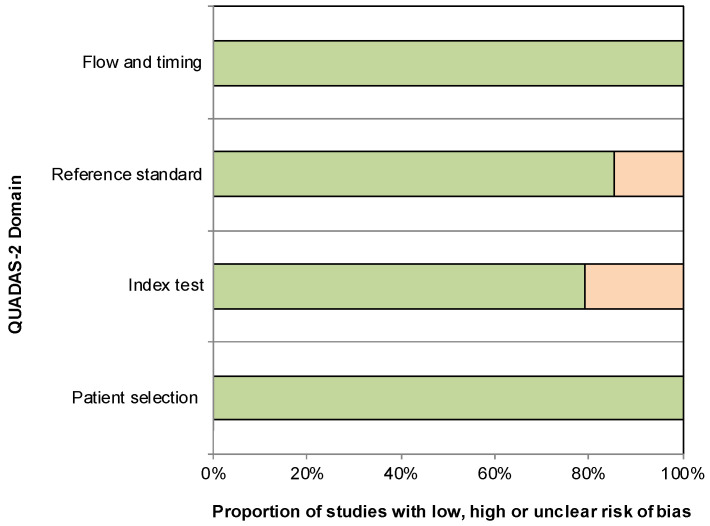
Assessment of methodological quality using QUADAS-2 tool. Green color indicates “low risk” studies and orange color indicates “high risk” studies.

Quality assessment results, including the proportions of studies with low and high risk of bias, are shown in Figure 2 (File S1). Regarding patient selection as well as flow and timing, all studies were rated as low risk. For reference standard, 7 out of 48 (15%) studies were classified as high risk because the location in which the tibial nerve CSA was measured was not precisely reported. For index test, 10 out of 48 (21%) studies were regarded as high risk because of the use of a single observer with no measurement of intra-observer reliability or because the expertise of the observers was not mentioned.

### 3.3. Demography of the Subjects

**Figure 3:**
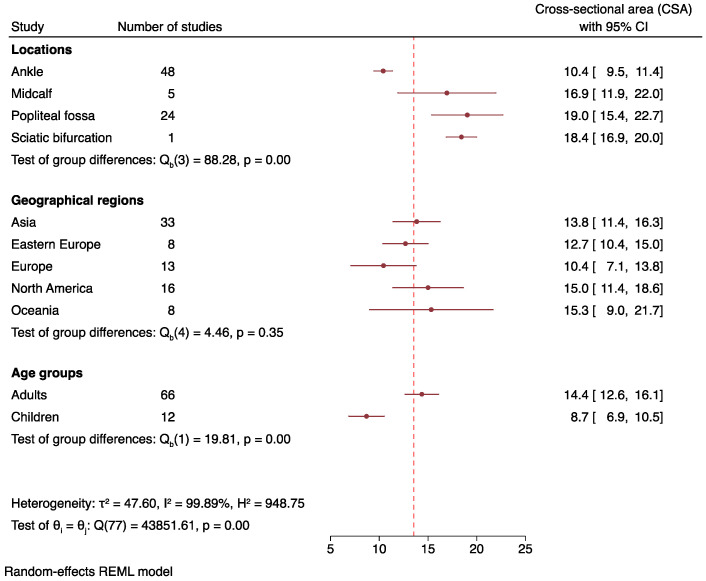
Cross-sectional area of tibial nerve in healthy subjects across subgroups.

Cohort characteristics of studies [4,5,6,7,10,11,13,14,15,16,17,23,24,25,26,27,28,29,30,31,32,33,34,35,36,37,38,39,40,41,42,43,44,45,46,47,48,49,50,51,52,53,54,55,56,57,58,59] included in the meta-analysis are summarized in Table 1. Forty-eight studies yielded a total of 2695 healthy subjects including 2503 (92.9%) adults and 192 (7.1%) children. Demographically, 1368 (50.8%) subjects were from Asia, 130 (4.8%) from Eastern Europe, 509 (18.9%) from Europe, 531 (19.7%) from North American and 157 (5.8%) from Oceania. Tibial nerve CSA was measured at four locations, including popliteal fossa (24 studies), sciatic nerve bifurcation (1 study), midcalf or mid-tibia (5 studies) and around the ankle (47 studies). Note that a single study may contain one to several groups of subjects categorized by location of CSA measurement. As a result, the total number of ultrasound studies (Figure 3) exceeded the total number of studies meeting the criteria for meta-analysis.

### 3.4. Overall Tibial Nerve Cross-Sectional Area

Pooled estimates of tibial nerve CSA in healthy subjects are depicted in Figure 3. In the popliteal fossa, the average tibial nerve CSA value was 19.0 mm^2^ (95% CI: 15.4–22.7, I^2^ = 99.28%). At mid-calf, the mean tibial nerve CSA was 16.9 mm^2^ (95% CI: 11.9–22.0, I^2^ = 99.62%). At the ankle, tibial nerve CSA was on average 10.4 mm^2^ (95% CI: 9.5–11.4, I^2^ = 99.68%). Subgroup analysis was further carried out to study the influence of age group, geographical region and BMI on tibial nerve CSA (Figure 3). Tibial nerve CSA was significantly larger (*p* < 0.01) in adults (14.4 mm^2^, 95% CI: 12.6–16.1, I^2^ = 99.91%) than in children (8.7 mm^2^, 95% CI: 6.9–10.5, I^2^ = 97.86%). Subgroup analysis by geographical region showed no statistically significant differences among the continents (*p* = 0.35). A funnel plot of effect sizes against standard errors were visually and statistically asymmetrical (*z* = 4.41, *p* < 0.01), suggesting the presence of publication bias (Figure S1). Leave-one-out meta-analysis was not performed because there were no potential outlier studies by observing the forest plot.

### 3.5. Tibial Nerve Cross-Sectional Area at the Popliteal Fossa and Ankle

**Figure 4:**
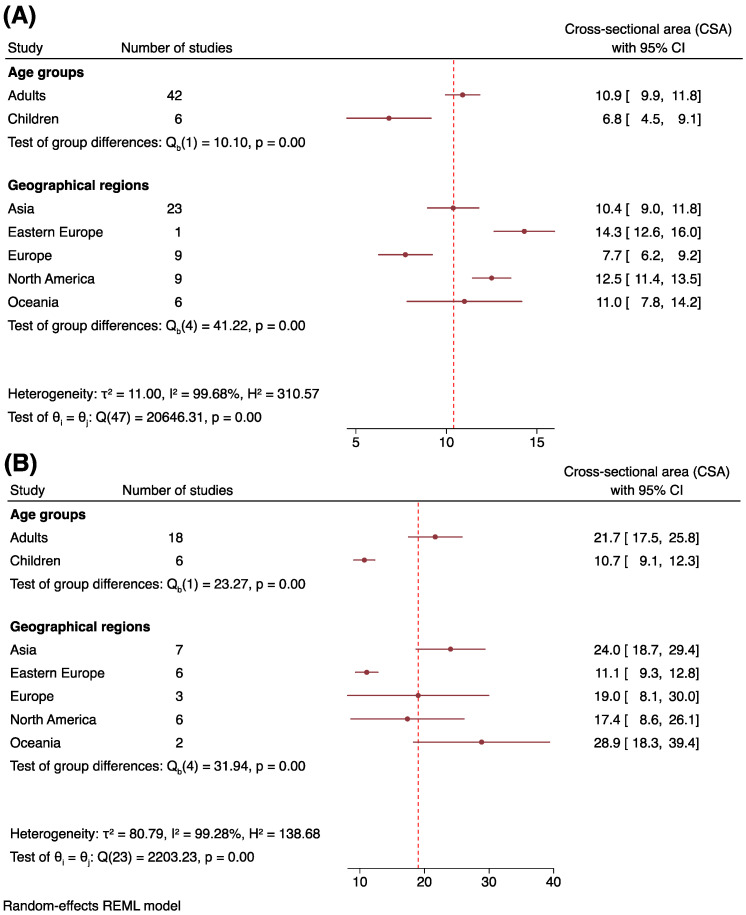
Cross-sectional area of the tibial nerve in healthy subjects and subgroup analysis at the ankle (**A**) and popliteal fossa (**B**).

Subgroup specific meta-analysis and regression analysis were carried out to study overall tibial nerve CSA at the ankle and popliteal fossa (Figure 4).

Analysis of 48 studies revealed that tibial nerve CSA at the ankle was on average 10.9 mm^2^ (95% CI: 9.9–11.8, I^2^ = 99.65%) in healthy adults and 6.8 mm^2^ (95% CI: 4.5–9.1, I^2^ = 98.72%) in healthy children (Figure 4A). Subgroup analysis showed statistically significant differences among the continents (*p* < 0.01) (Figure 4A), with Eastern Europeans demonstrating the largest tibial nerve CSA (14.3 mm^2^, 95% CI: 12.6–16.0), while Europeans had the smallest tibial nerve CSA (7.7 mm^2^, 95% CI: 6.2–9.2). Similar to the overall tibial nerve CSA analyzed previously, the funnel plot was significantly asymmetrical, indicating publication bias (*z* = 3.16, *p* < 0.01) (Figure S2). Leave-one-out meta-analysis was conducted to detect the influence of one potential outlier study [44]. The results show that the overall tibial nerve CSA did not change significantly (Figure S3). Correlation between tibial nerve CSA and distance relative to the medial malleolus was analyzed (0 to 7 cm proximal to the medial malleolus) and was not found to be statistically significant (Pearson’s *r* = −0.31, *p* = 0.07) (Figure S4).

At the popliteal fossa, analysis of 24 studies indicated that the overall tibial nerve CSA was 21.7 mm^2^ (95% CI: 17.5–25.8, I^2^ = 99.35%) in adults and 10.7 mm^2^ (95% CI: 9.1–12.3, I^2^ = 68.05%) in children (Figure 4B). Subgroup analysis revealed statistically significant differences among the continents (*p* < 0.01) (Figure 4B), with Oceanians demonstrating the largest tibial nerve CSA value, followed by Europeans, Asians, North Americans, and Eastern Europeans. The funnel plot was symmetrical (*z* = 0.55, *p* = 0.58), suggesting the absence of publication bias. Leave-one-out meta-analysis was not performed because no potential outlier study was identified.

**Figure 5:**
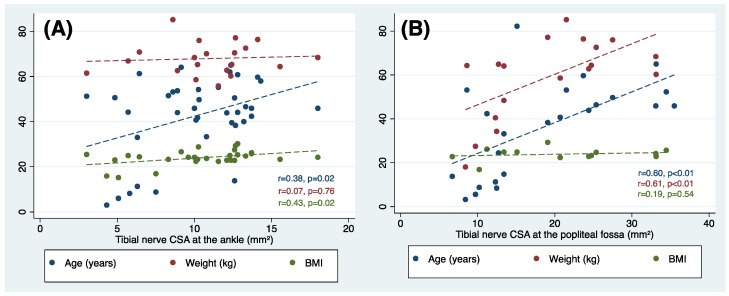
Correlation between tibial nerve cross-sectional and age, weight or body mass index in healthy subjects and the ankle (**A**) and popliteal fossa (**B**).

Regression analysis was then performed to evaluate the effect of age, weight and BMI on the mean CSA at the ankle (Figure 5A) and popliteal fossa (Figure 5B). The tibial nerve CSA increased significantly with age at both at the ankle (Pearson’s *r* = 0.38, *p* = 0.02) and the popliteal fossa (Pearson’s *r* = 0.60, *p* < 0.01). Tibial nerve CSA was positively associated with weight only at the popliteal fossa (Pearson’s *r* = 0.61, *p* < 0.01). No correlations were found for the BMI at both sites (Figure 5).

## 4. Discussion

In the present study, we established the normative reference values of tibial nerve CSA in healthy individuals. Although a recent systematic review and meta-analysis [12] has provided reference values of tibial nerve CSA in healthy individuals based on 16 ultrasonographic studies, we note that a substantial amount of literature may be missing because only studies in healthy individuals were included. Healthy controls of other potential studies, however, were inadvertently neglected. To include a broader range of studies, we intentionally omitted the keyword ‘normal’ from our systematic review. As a result, we identified a total of 48 studies involving 2695 individuals, nearly four times the number analyzed in the previous meta-analysis [12].

We found that the tibial nerve CSA was the largest at the popliteal fossa, followed by mid-calf level and the ankle. However, the results exhibit significant between-study heterogeneity, with I^2^ values exceeding 99% for tibial nerve CSA across all three anatomical sites. Therefore, subgroup analysis and regression analysis were attempted to discern the causes of heterogeneity and the variables that might mediate tibial nerve CSA (Figure 3). Subgroup analysis was performed only at the ankle and popliteal fossa (Figure 4) because a number of studies performed at the other sites were insufficient. Since significant between-study heterogeneity persisted even after subgroup analysis, we conducted correlational analysis to examine the potential impact of other factors, such as age, weight, and BMI, on tibial nerve CSA.

The effect of age on tibial nerve CSA has been controversial. Eight previous studies [13,16,17,24,25,47,52,59] have confirmed a significant influence of age, while four studies [9,15,16,26] found no correlation. In this study, we found that the effect of age was statistically significant (Figure 5). This result is in contrast with our previous meta-analysis of tibial nerve CSA in diabetic patients, in which the tibial nerve CSA in healthy control groups was relatively consistent across all age groups [60]. The significant effect of age could be the result of normal somatic growth of the nerve, which reaches a climax during late childhood or early adulthood [51]. The absence of a correlation with age in our previous study [60] is probably due to the use of matched-control patients; as a result, no data from children were obtained. Studies have reported divergent findings regarding the impact of height, weight, and BMI, as reported by Singh et al. [10]. We did not analyze height in the present study due to its heterogeneity, especially in children of a similar age [51]; instead, we focused solely on weight and BMI. In this study, we found a significant correlation between tibial nerve CSA weight at the popliteal fossa but not the ankle. The impact of weight was also confirmed by 12 other studies [10,13,15,16,23,24,25,26,46,47,52,59], while only two studies found no such correlation [7,17]. Though the effect of height was not studied in this meta-analysis, eight studies [10,15,16,23,46,47,52,59] reported a significant impact of height, while only four studies [7,13,17,26] found no correlation. Finally, we note there is no standardized protocol when measuring the tibial nerve CSA at the ankle and the probe could be placed anywhere from 0 to 7 cm proximal to the medial malleolus (Table 1). Because of this, the correlation between tibial nerve CSA and distance relative to the medial malleolus was analyzed. Although the correlation was not statistically significant, we observed that tibial nerve CSA tended to be higher when measured at 5 cm or more above the medial malleolus, while it remained fairly uniform between 0–5 cm above the medial malleolus (Figure S4). This result is consistent with the findings of Ranjan et al. [61] who have reported that tibial nerve CSA did not change much in the same person at 1, 3 or 5 cm above the medial malleolus. Another factor that might cause high between-study heterogeneity is ankle position during the CSA measurement. It was found that tibial nerve CSA differed significantly depending on the ankle position [62].

By establishing normative reference values of tibial nerve CSA, a cut-off point can be used as a clinical tool to detect patients with suspected chronic neuropathies. Tibial nerve CSA was found to be elevated in patients with tarsal tunnel syndrome [33,59]. Recently, Senarai et al. [60] have observed that tibial nerve CSA was statistically larger in diabetic patients with peripheral neuropathy when compared with baseline diabetic patients or healthy controls. Nerve swelling in diabetic patients with neuropathy strongly correlated with chronic inflammation, hyperglycemia, and other risk factors associated with diabetes [63].

Several conditions can result in nerve swelling, including leprosy, hereditary motor and sensory neuropathies, and chronic inflammatory demyelinating neuropathies [64]. The pathophysiology of nerve enlargement involves the proliferation of periaxonal Schwann cells, resulting in nerve thickening resembling an onion bulb-like structure, ultimately leading to chronic recurrent demyelination of the nerve. Additionally, factors such as blood vessels, cells, and certain agents within the endoneurium can also contribute to an increase in nerve CSA [65].

Because of methodological differences, between-study heterogeneity, and other confounding variables, establishing cut-off values for tibial nerve CSA in these three groups was not possible. This limits the clinical usefulness of tibial nerve CSA as a diagnostic marker for diabetic peripheral neuropathy [60]. For screening and diagnosing diabetic peripheral neuropathy, a cut-off value in healthy individuals reported in this study might be equally useful. Likewise, tibial nerve CSA was also found to be high in patients with tarsal tunnel syndrome [33,59]. Tibial nerve CSA in tarsal tunnel syndrome patients was investigated by Tawfik et al. [59]. The CSA value at 19 mm^2^, with a sensitivity of 61% and a specificity of 88% was proposed as a cut-off value for healthy tibial nerves. In this study, the average tibial nerve CSA in healthy adults was 10.9 mm^2^ (95% CI: 9.9–11.8). In our opinion, the upper limit of 11.8 mm^2^ could serve as a cut-off point for healthy tibial nerves, with values higher than this possibly requiring further investigation. This cut-off point may be applicable as a screening tool to early detect diabetic peripheral neuropathy, preempting the stage of permanent limb damage or even amputation. In addition, since tibial nerve CSA at the ankle was strongly correlated with age and BMI even in healthy individuals, these two factors should always be considered when studying tibial nerve CSA.

## 5. Limitations

The present work is not without limitations. The majority of the included patients were Asians, which introduces a potential bias towards a single population group. While the funnel plot and associated statistical analysis suggested the possibility of moderation by publication bias or small study effects, plot asymmetry could also be attributed to other factors, such as heterogeneity [66]. High between-study heterogeneity was observed, so the results of this meta-analysis should be interpreted with caution. Apart from the sub-variables that were studied by subgroup analysis or meta-regression, possible causes of heterogeneity may be observer- and machine-dependent, such as the protocol used to evaluate tibial nerve CSA and the difference in the transducers used. It was observed that selecting different neural brightness settings could lead to varying CSA values [10], highlighting the need for standardized research methodology. Among the 48 included studies, only a small number mentioned the statistical tools used for testing inter- or intra-observer reliability. Correlations with height, sex difference and side difference were not analyzed due to insufficient data.

## 6. Conclusions

This study established the normative reference values of the tibial nerve CSA in healthy subjects. Among the key results, we found that tibial nerve CSA in healthy adults should not exceed 11.8 mm^2^ at the ankle and is positively correlated with age and BMI. The establishment of normal reference values for tibial nerve CSA is valuable for preliminary screening of tibial nerve neuropathy such as in patients with diabetic peripheral neuropathy or tarsal tunnel syndrome. In cases of diabetic neuropathy, we believe that ultrasound serves as a valuable screening tool for the early detection of tibial nerve abnormalities, preempting the stage of irreversible sensory loss which could lead to permanent damage. Nevertheless, electromyography, nerve conduction studies and sensation studies remain the gold standard when diagnosing these neuropathies. Further research is still needed to study tibial nerve CSA in wider ethnic populations using a more standardized methodology that can be adapted universally to avoid extreme between-study heterogeneity.

## Figures and Tables

**Table 1:** Cohort characteristics of 48 studies included in this meta-analysis.

Study	Year	Country	Location	Subjects ^1^	CSA	SD	Age	Weight	BMI
Bae and An [23]	2022	South Korea	Popliteal fossa	107	24.73	6.03	N/A	64.4	23.3
			Midcalf	107	12.97	3.71	N/A	64.4	23.3
			Ankle	107	15.55	3.8	N/A	64.4	23.3
Bedewi et al. [24]	2018	Saudi Arabia	Ankle	138	12.66	4.45	38.33	77.15	29.31
			Popliteal fossa	138	19.08	6.88	38.33	77.15	29.31
Bedewi et al. [25]	2021	Saudi Arabia	Popliteal fossa	72	13.4	3.4	33.2	64.1	24.9
Boehm et al. [26]	2014	Hungary and Germany	Ankle	56	9.6	2.2	N/A	N/A	24.2
Borire et al. [27]	2018	Australia	Ankle	30	12.8	0.5	60.8	N/A	30.2
Boyd and Dilley [28]	2014	USA	Ankle	20	13.32	1.53	46.6	72.59	24.75
			Popliteal fossa	20	25.37	4.41	46.4	72.59	24.75
Breiner et al. [29]	2016	Canada	Ankle	100	12.8	3.5	44.1	N/A	25.3
Cartwright et al. [16]	2008	USA	Midcalf	60	25.3	7.3	45.9	N/A	N/A
			Popliteal fossa	60	35.5	10.3	45.9	N/A	N/A
			Ankle	60	13.7	4.3	45.9	N/A	N/A
Cartwright et al. [14]	2013	USA	Popliteal fossa	60	11.2	3.3	42.4	N/A	26.2
			Popliteal fossa	12	15.1	5.5	82.2	N/A	24.9
			Ankle	5	12.6	2.1	13.8	N/A	22.8
			Ankle	60	13.7	4.3	42.4	N/A	26.2
			Popliteal fossa	3	6.7	3.1	13.8	N/A	22.8
			Popliteal fossa	4	10.2	2.9	8.8	N/A	16.9
			Ankle	4	7.5	2.5	8.8	N/A	16.9
Chen et al. [30]	2000	China	Ankle	33	8.31	2.32	51.51	N/A	23.28
Dikici et al. [31]	2017	Türkiye	Ankle	20	14.3	3.8	58	N/A	N/A
Druzhinin et al. [13]	2019	Russia	Popliteal fossa	7	9.71	2.82	5.61	27.5	N/A
				5	12.46	1.69	8.47	34.3	N/A
				7	12.3	3.82	11.3	40.5	N/A
				22	12.7	8.3	24.5	64.9	N/A
				4	13.4	6.27	14.8	48.4	N/A
				12	8.41	2.39	3.27	18.1	N/A
Fantino et al. [33]	2021	France	Midcalf	21	10.6	1.8	39	67	N/A
Elfattah Hassan Gadalla et al. [32]	2022	Egypt	Ankle	20	13.2	3.1	40	N/A	N/A
Garg et al. [34]	2018	Australia	Ankle	17	14.8	3.2	N/A	N/A	N/A
Goyal et al. [35]	2021	India	Ankle	70	5.7	1.3	44.2	66.9	24.95
Grimm et al. [58]	2014	Germany	Ankle	21	8.6	2.7	53.14	85.2	N/A
			Popliteal fossa	21	21.5	4.4	53.14	85.2	N/A
Grimm et al. [58] ^2^	2014	Germany	Ankle	8	10.3	2.5	49.71	76	N/A
			Popliteal fossa	8	27.5	7	49.71	76	N/A
			Popliteal fossa	21	8.6	2.7	53.14	64.29	N/A
Grimm et al. [36]	2018	Germany	Ankle	100	10.2	2	N/A	N/A	N/A
He et al. [37]	2019	China	Ankle	40	11.55	1.59	55.2	55.83	22.38
Hobbelink et al. [38]	2018	Australia	Ankle	5	5.8	0.9	8.2	N/A	N/A
Hooper et al. [39]	2011	Canada	Ankle	32	10.78	1.72	33.3	70.1	23.7
Ibrahim [40]	2022	Egypt	Ankle	50	10.26	1.86	54.23	N/A	28.81
Ishibashi et al. [4]	2016	Japan	Ankle	29	4.84	0.16	50.6	N/A	23
Issar et al. [41]	2022	Australia	Ankle	28	12.3	3.1	62	N/A	25
Ito et al. [42]	2007	Japan	Ankle	35	7.9	1.5	N/A	N/A	N/A
Jain et al. [43]	2009	India	Ankle	30	6.3	3.2	33	N/A	N/A
Jang et al. [44] ^3^	2014	South Korea	Ankle	18	10	1.5	45.9	68.4	24.2
				18	18	4	45.9	68.4	24.2
Jang et al. [44]	2014	South Korea	Popliteal fossa	18	33.1	3.8	45.9	68.4	24.2
Kang et al. [5]		South Korea	Ankle	20	12.36	2.85	65	60.25	22.86
	2016		Popliteal fossa	20	33.14	4.92	65	60.25	22.86
			Midcalf	20	16.39	2.95	65	60.25	22.86
Kelle et al. [6]	2016	Turkey	Sciatic bifurcation	53	18.43	5.79	57.8	N/A	30.22
Kerasnoudis et al. [7]	2013	Germany	Ankle	75	6.36	1.45	N/A	N/A	N/A
Lothet et al. [46]	2019	USA	Ankle	140	13.7	4.3	N/A	N/A	25.8
Niu et al. [47]	2021	China	Ankle	111	10.2	1.9	41.7	65.3	23.3
Noto et al. [48]	2018	Australia	Popliteal fossa	30	23.7	7.4	59.7	76.4	N/A
			Ankle	30	14.1	3.2	59.7	76.4	N/A
Pelosi et al. [57]	2022	New Zealand	Popliteal fossa	18	34.46	11	52.3	N/A	25.7
Pitarokoili et al. [49]	2016	Germany	Ankle	55	9.14	2.41	64.1	N/A	26.64
Qrimli et al. [17]	2016	Canada	Ankle	98	12.7	3.1	N/A	N/A	N/A
Razali et al. [50]	2016	Malaysia	Ankle	17	12.6	5.4	50.5	70.5	27.5
Schubert et al. [51]	2020	Germany	Ankle	57	5.07	1.51	6	N/A	15.2
				57	4.31	1.38	3	N/A	15.9
Seok et al. [15]	2014	South Korea	Ankle	94	12.1	3.1	43.9	62.8	22.9
			Popliteal fossa	94	24.4	4.4	43.9	62.8	22.9
Sindhu et al. [52]	2022	India	Ankle	100	10.1	2.23	40.7	58.6	22.41
			Popliteal fossa	100	20.7	4.41	40.7	58.6	22.41
Singh et al. [9]	2017	India	Ankle	75	12.42	1.1	39.54	65.34	N/A
Singh et al. [10]	2022	India	Midcalf	200	19.6	1.4	N/A	N/A	N/A
			Ankle	200	11.1	1.1	N/A	N/A	N/A
Sreejith et al. [53]	2021	India	Ankle	30	8.9	2.319	44	N/A	N/A
Tandon et al. [54]	2021	India	Ankle	30	3.01	0.61	51.26	61.5	25.46
Tawfik et al. [59]	2016	Egypt	Ankle	17	13.8	4.4	N/A	N/A	N/A
van Maurik et al. [55]	2014	Netherlands	Ankle	38	6.43	1.32	61.29	70.84	24.4
Watanabe et al. [11]	2010	Japan	Ankle	32	8.9	2.8	53.7	62.6	N/A
Yiu et al. [56]	2015	Australia	Ankle	29	6.3	1.9	11.3	N/A	N/A

^1^ Total number of subjects in this column exceeded 2695 because several measurements at different sites were made for some studies. ^2^ There were two studies by Grimm et al., 2014 [58]. ^3^ This study performed two measurements at different levels of the ankle.

## Data Availability

The data that support the findings of this study are available from the corresponding author upon reasonable request.

## Supplementary Materials

The following supporting information can be downloaded at: https://www.mdpi.com/article/10.3390/jcm12196186/s1, Figure S1: funnel plot of overall CSA; Figure S2: funnel plot of CSA at the ankle; Figure S3: leave-one-out meta-analysis; Figure S4: Correlation between tibial nerve CSA and distance relative to the medial malleolus; File S1: raw data for meta-analysis.
