# Supplementary figures and images for: Normative Reference Values of the Tibial Nerve in Healthy Individuals Using Ultrasonography: A Systematic Review and Meta-Analysis
Source: J Clin Med. 2023 Sep 25;12(19):6186. doi: 10.3390/jcm12196186 (PMC10573196; doi:10.3390/jcm12196186)

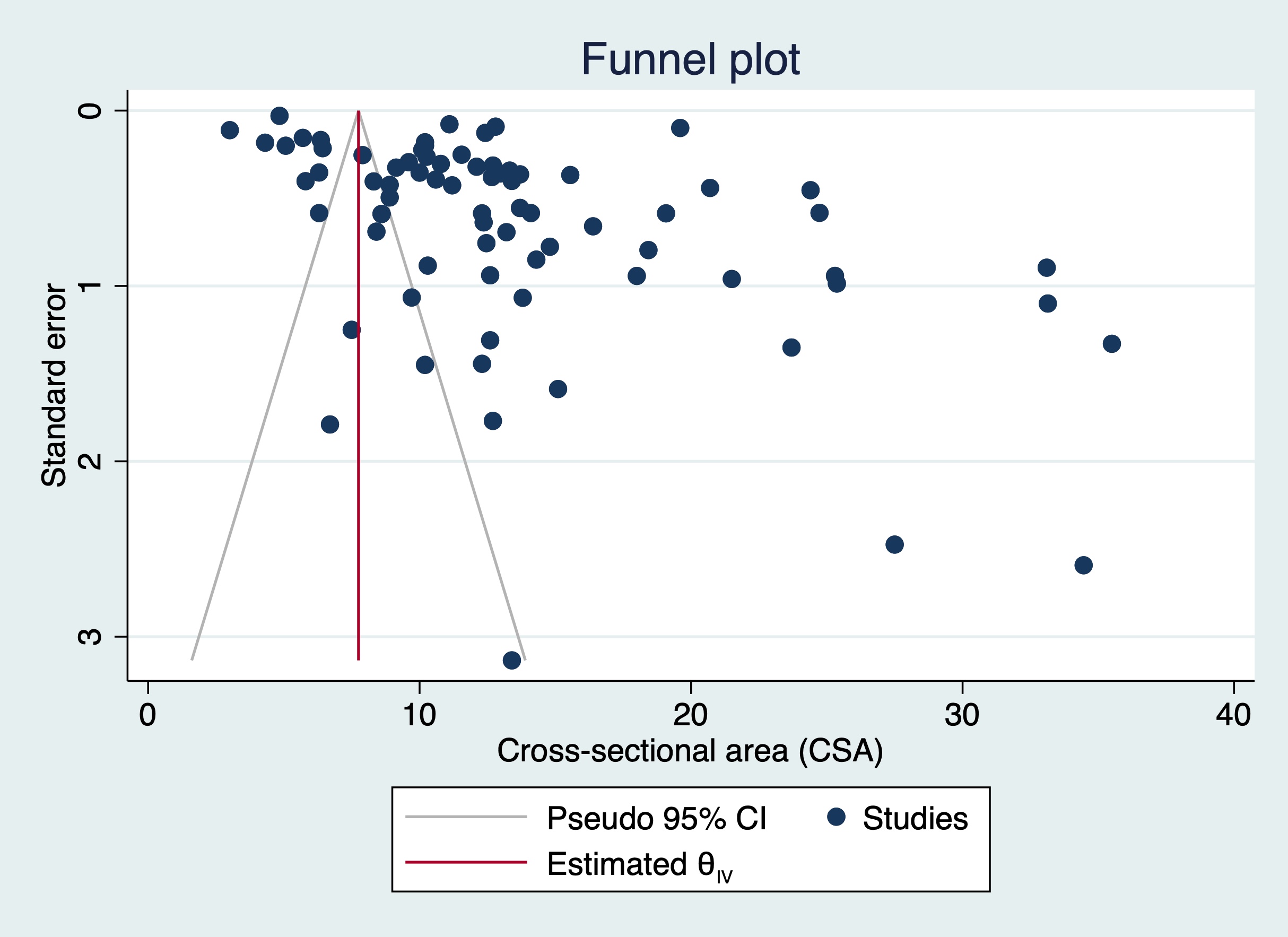

Supplement: Supplementary file 1 [file jcm-12-06186-s001.zip › Figure S1.jpg]

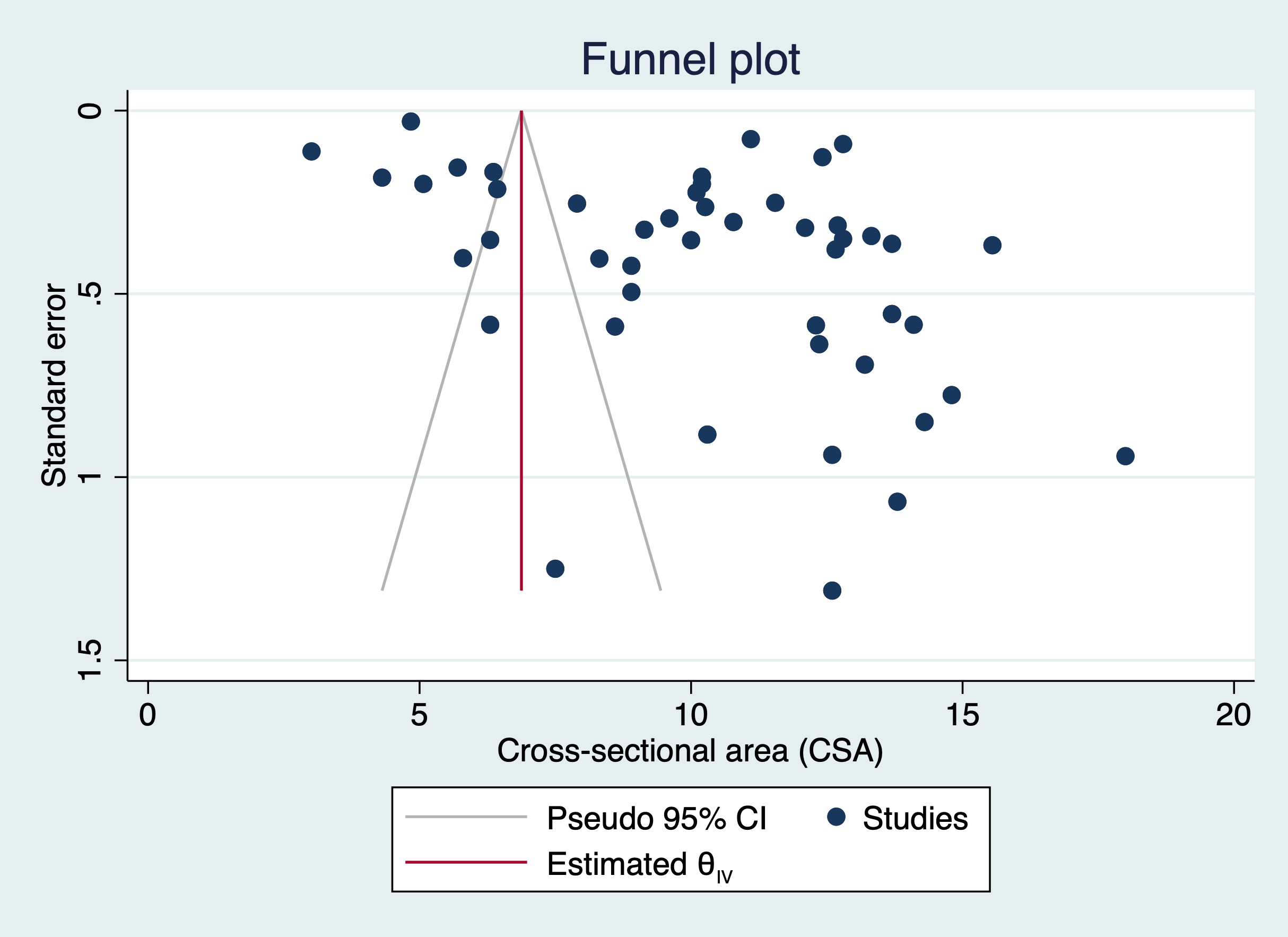

Supplement: Supplementary file 1 [file jcm-12-06186-s001.zip › Figure S2.jpg]

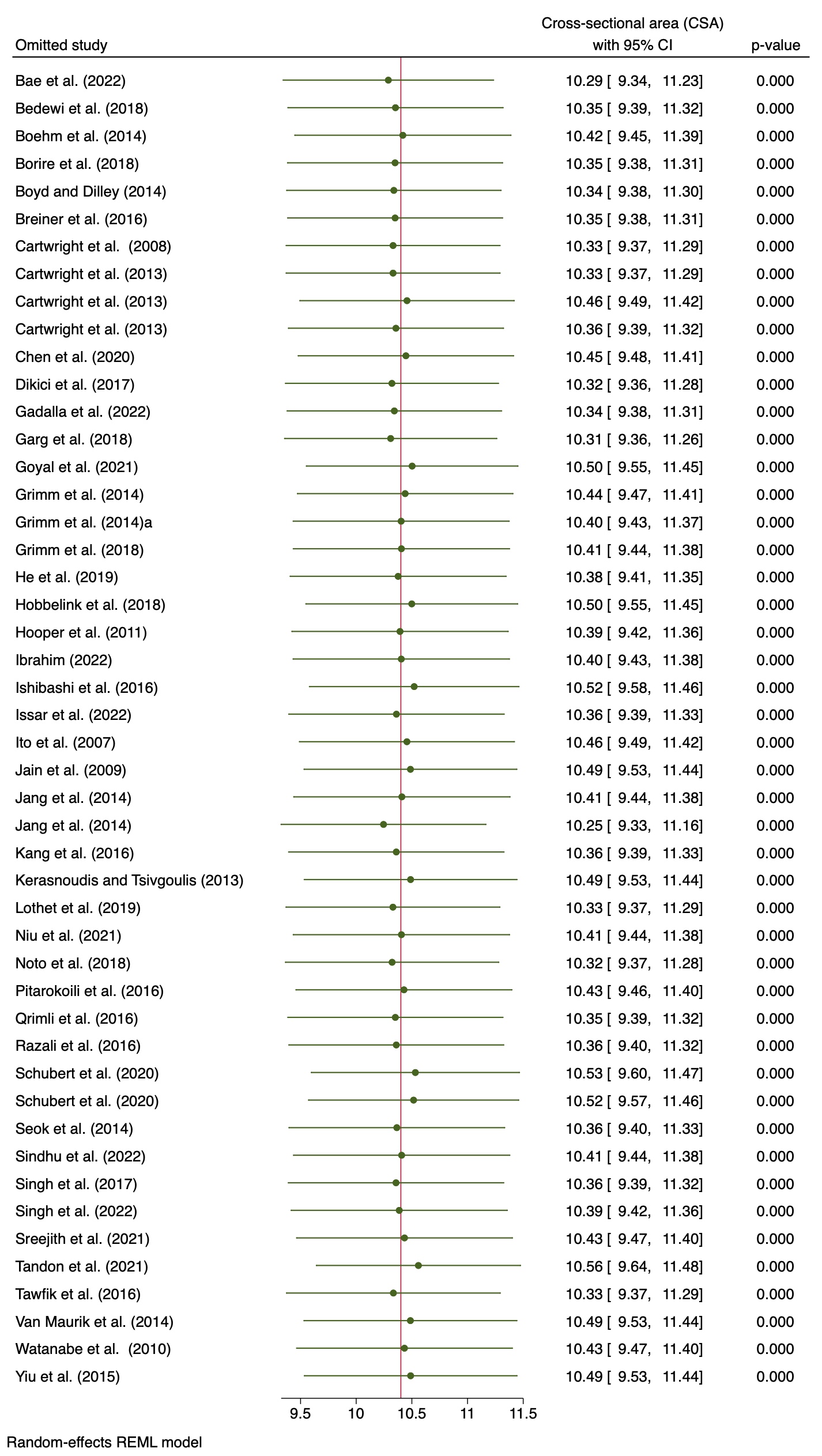

Supplement: Supplementary file 1 [file jcm-12-06186-s001.zip › Figure S3.jpg]

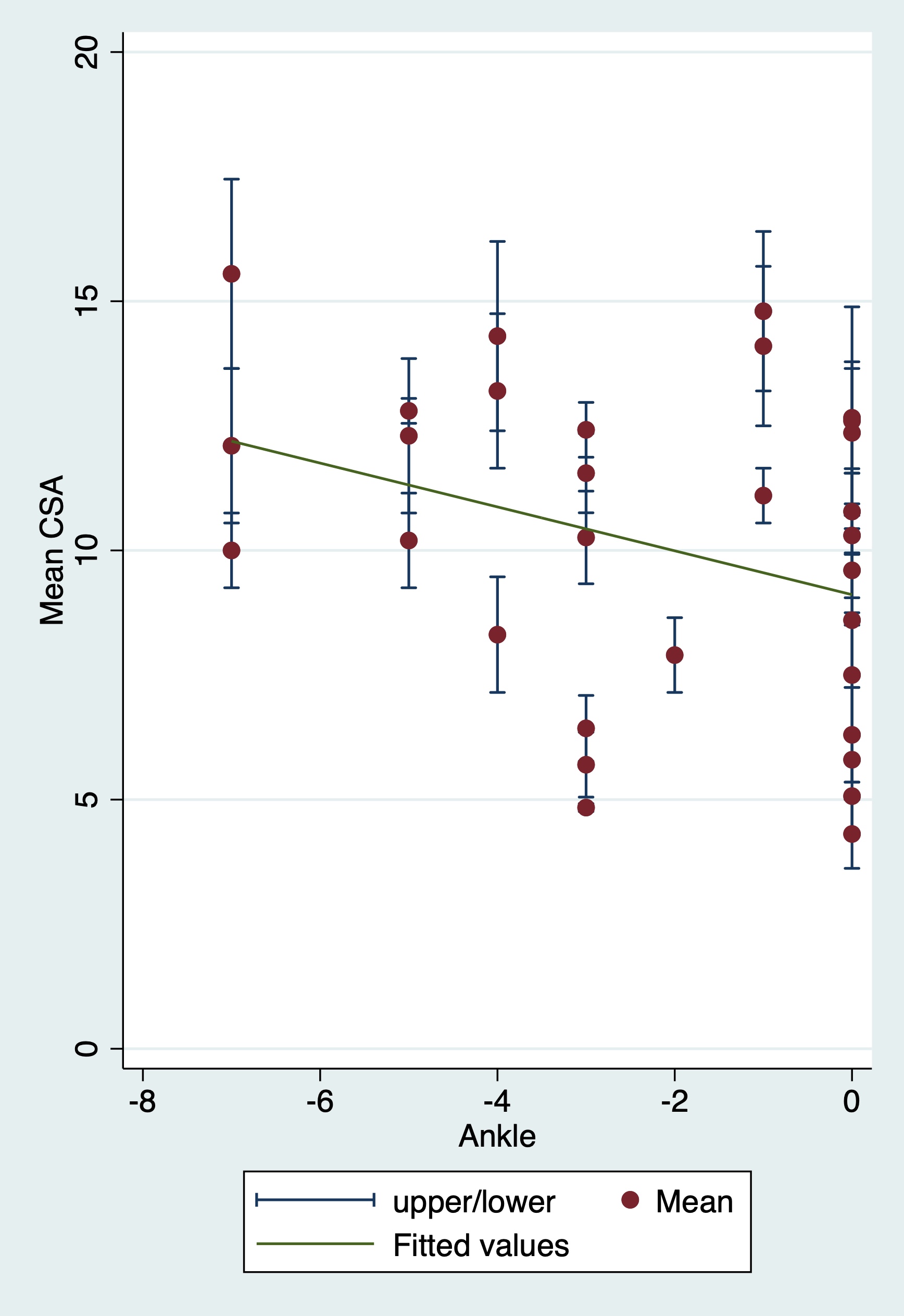

Supplement: Supplementary file 1 [file jcm-12-06186-s001.zip › Figure S4.jpg]
